# Does body composition matter in patients with systemic sclerosis?

**DOI:** 10.1093/rheumatology/keaf283

**Published:** 2025-05-23

**Authors:** Chiara Giraudo, Giulia Fichera, Marco Binda, Beatrice Moccaldi, Elisabetta Cocconcelli, Anna Cuberli, Anna Michielin, Andrea Doria, Roberto Stramare, Elisabetta Balestro, Elisabetta Zanatta

**Affiliations:** Unit of Advanced Clinical and Translational Imaging, Department of Cardiac, Thoracic, Vascular Sciences and Public Health, Padova University Hospital, Padova, Italy; Unit of Advanced Clinical and Translational Imaging, Department of Cardiac, Thoracic, Vascular Sciences and Public Health, Padova University Hospital, Padova, Italy; Rheumatology Unit, Department of Medicine—DIMED, Padova University Hospital, Padova, Italy; Rheumatology Unit, Department of Medicine—DIMED, Padova University Hospital, Padova, Italy; Respiratory Disease Unit, Department of Cardiac, Thoracic, Vascular Sciences and Public Health, Padova University Hospital, Padova, Italy; Rheumatology Unit, Department of Medicine—DIMED, Padova University Hospital, Padova, Italy; Unit of Advanced Clinical and Translational Imaging, Department of Cardiac, Thoracic, Vascular Sciences and Public Health, Padova University Hospital, Padova, Italy; Rheumatology Unit, Department of Medicine—DIMED, Padova University Hospital, Padova, Italy; Unit of Advanced Clinical and Translational Imaging, Department of Cardiac, Thoracic, Vascular Sciences and Public Health, Padova University Hospital, Padova, Italy; Respiratory Disease Unit, Department of Cardiac, Thoracic, Vascular Sciences and Public Health, Padova University Hospital, Padova, Italy; Rheumatology Unit, Department of Medicine—DIMED, Padova University Hospital, Padova, Italy; Department of Cardiac, Thoracic, Vascular Sciences and Public Health, Padova University Hospital, Padova, Italy

**Keywords:** SSc, body composition, CT, imaging

## Abstract

**Objectives:**

Body composition plays a significant role in various rheumatic and autoinflammatory diseases. The aim of our study was to assess the impact of muscle mass and subcutaneous adipose tissue quality and quantity in patients with SSc.

**Methods:**

Adults with SSc referring to our tertiary centre who underwent high-resolution chest CT (HRCT) to assess pulmonary involvement were included. A semi-automatic segmentation of the subcutaneous fat and paravertebral muscle was performed at the level of the 12th dorsal vertebra, and body composition metrics were collected (subcutaneous fat and paravertebral muscle area and density). Stepwise linear regression analysis was applied to evaluate if body composition, demographics and pulmonary function tests acted as predictors of mortality. Considering patients with muscle Hu values <30 as affected by myosteatosis, we used the odds-risk ratio to assess if it is associated with a higher risk of mortality.

**Results:**

Eighty-seven SSc patients (77 females, age 60 ± 15 years, 61% affected by limited cutaneous SSc) were included. The linear model demonstrated that lower DLCO (*P* = 0.047), higher BMI (*P* = 0.013), higher density of the subcutaneous fat (*P* = 0.005) and lower skeletal muscle index (*P* < 0.001) acted as predictors of mortality. Overall, 63 patients (72%) were affected by myosteatosis (i.e. Hu <30 Hu) and patients affected by muscle fat infiltration at CT showed a 3.345 times higher mortality risk (95% CI 0.396–28.295).

**Conclusion:**

Patients with SSc are affected by myosteatosis and pre-sarcopenia and body composition seems to influence the overall outcome.

Rheumatology key messages

- Patients with SSc are frequently affected by myosteatosis at imaging.
- SSc patients affected by muscle fat infiltration on CT scans have higher mortality risk.
- Body composition assessment may help predict the overall outcome in SSc.

## Introduction

In the last years, changes in body composition, including sarcopenia, sarcopenic obesity and cachexia, drew high interest in the scientific community [1, 2]. Indeed, it has been demonstrated that they have a strong impact on the outcome of various diseases [1, 2]. Regarding sarcopenia, still much has to be done to achieve unanimous diagnostic criteria and in terms of imaging, so far, only the role of dual-energy X-ray absorptiometry (DXA) has been recognized by international working groups [3]. Despite the lack of assessment of muscle strength via imaging, various techniques, beyond DXA, provide crucial information about muscle quantity and quality as well as on the evaluation of adiposity. Indeed, it has been shown that body composition metrics collected by CT act as a predictor of outcome in various types of tumours [1, 2]. Even if most of the literature was initially focused on oncological patients, recently the role of body composition has been investigated also for inflammatory and autoimmune conditions [4, 5]. Since inflammatory cytokines contribute to muscle loss and increased adiposity, in patients with inflammatory disease, a synergistic action may occur. For example, sarcopenia affects around 33% of patients with RA and 25% of those with spondyloarthritis [4, 5].

Despite this growing evidence, to the best of our knowledge, the impact of body composition on SSc has not been investigated by imaging yet. In this rare autoimmune disease, primarily affecting the skin and internal organs, with pulmonary disease, expressed as interstitial lung disease (ILD) and/or pulmonary artery hypertension, being the leading cause of mortality [6], the musculoskeletal involvement is poorly characterized. Given the systemic involvement typical of SSc, we explored the role of body composition and muscle status in this group of patients using CT.

## Methods

Patients with SSc (EULAR/ACR 2013 criteria) referring to our tertiary centre from January 2014 to January 2023 who underwent a HRCT scan to assess pulmonary involvement were included in this retrospective, Institution Review Board-approved (Azienda Ospedale Università di Padova, Padova, Italy, 5505/AO/22) study [7]; given the retrospective study design and the analysis of anonymized clinical data, informed consent for publication was waived.

**Figure 1.**
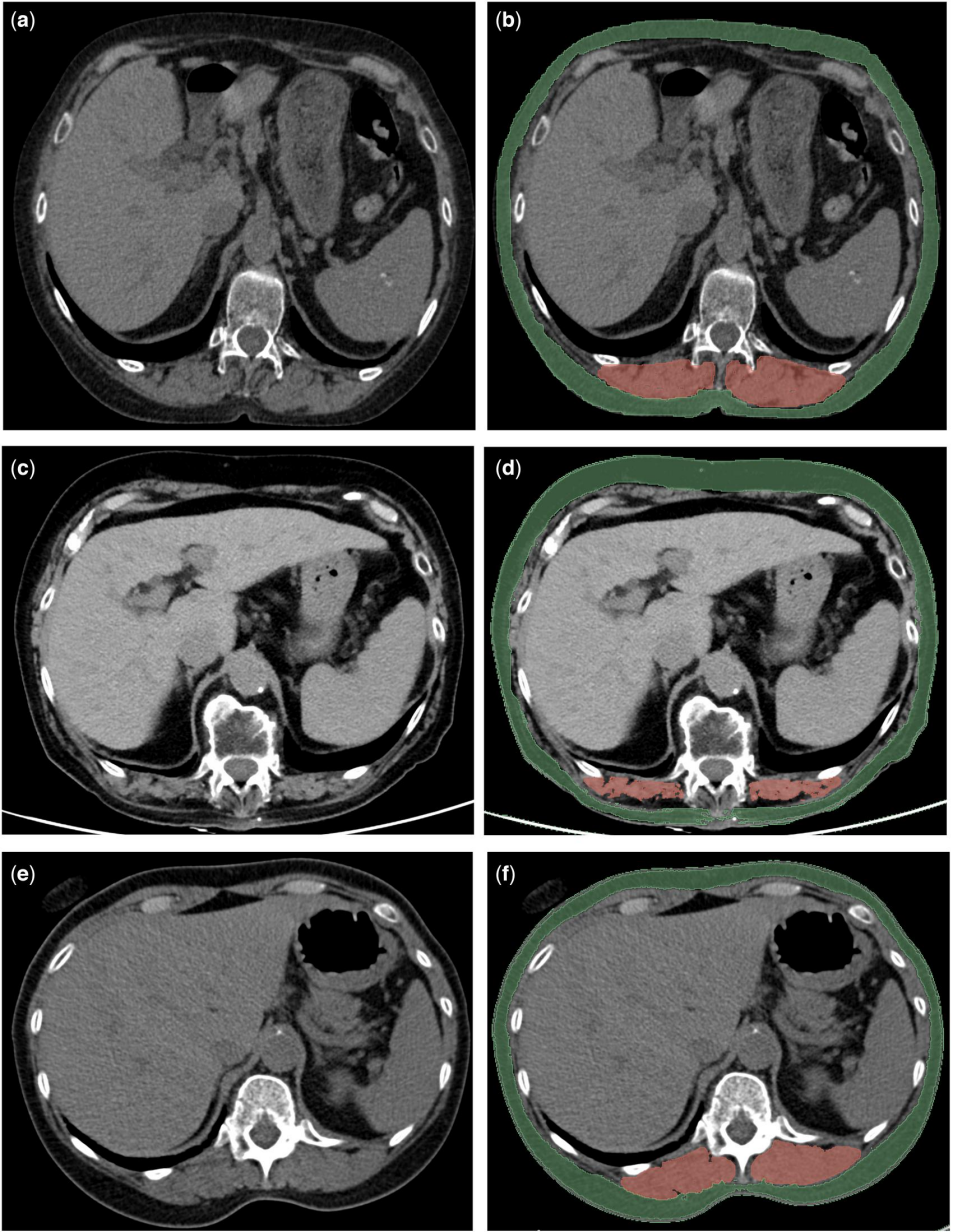
Axial mediastinal window of the high-resolution CT of a 75-year-old female patient with SSc at the level of the 12th thoracic vertebra, used for the body composition evaluation (**a**). In **b**, representation of the applied segmentation of the paravertebral muscle (red) and of the subcutaneous fat (green). Axial mediastinal window of the high-resolution CT of a 79-year-old female with limited SSc (lcSSc) at the level of the 12th thoracic vertebra (**c**) with muscle and subcutaneous segmentation (respectively red and green areas in **d**) nicely shows the reduced muscle mass and the myosteatosis. Equivalent mediastinal window with the corresponding segmentation of a 54-year-old female with diffuse SSc (dcSSc) (**e** and **f**), showing a larger muscle area with preserved muscle density. Moreover, the patient with lcSSc had more hypodense fat tissue (−101 Hu) than the patient with dcSSc (−92 Hu)

For each patient, a semi-automatic segmentation of the paravertebral muscles and subcutaneous tissue was performed at the level of the upper plate of the 12th thoracic vertebra using the soft tissue window and applying standardized thresholds (−29 to 150 Hounsfield Unit, Hu, and −30 to −190 Hu, respectively, for muscle and fat) (Fig. 1a and b); then muscle and subcutaneous area and density were extracted. The skeletal muscle index (SMI) was computed by applying the formula: skeletal muscle cross-sectional area (cm^2^)/height^2^ (m^2^) [8].

The following variables were collected: demographics (age and gender), height (cm), weight (kg), BMI, disease duration (months from the first non-Raynaud sign/symptom), the modified Rodnan skin score (mRSS), the European Scleroderma Trials and Research Group activity index (EUSTAR-AI), ongoing and/or previous treatment with steroids (yes/no) for at least 2 years. In our centre, steroids are prescribed in SSc patients with the ‘inflammatory phenotype’ (diffuse cutaneous form and musculoskeletal manifestations), while following the guidelines of EULAR, immunosuppressants are used for pulmonary fibrosis, musculoskeletal and diffuse skin involvement [9]. Organ involvement—scleroderma renal crisis, ILD, gastrointestinal and cardiac involvement—was also recorded. The autoantibody profile (antinuclear antibody (ANA), anti-Scl-70, anti-centromere, anti-RNA polymerase III and anti-PM/Scl) was also recorded, and patients were classified into limited (lc) or diffuse cutaneous (dc) SSc [7].

Pulmonary function tests, including FVC (forced vital capacity), TLC (total lung capacity) and DLCO (diffusion lung carbon monoxide), were performed with CareFusion MasterScreen™ PFT within 3 months before/after the HRCT.

In case of ILD, the following patterns were reported: usual interstitial pneumonia, non-specific interstitial pneumonia (NSIP), organizing pneumonia; patients with atypical features were considered as affected by ILD but not included in any specific category.

### Statistical analysis

Descriptive statistics were performed. Stepwise linear regression analysis was used to evaluate if any body composition, demographic, clinical and laboratory variables as well as functional respiratory parameters acted as predictors of mortality [10–12].

The independent samples *t* test was applied to investigate any potential difference in terms of body composition metrics (Hu and area of muscle and subcutaneous tissue) between patients with lcSSc and dcSSC; moreover, it was applied to assess if there was any difference in age (years) and disease duration (months) between patients with or without myosteatosis. Regarding this last aspect, in case of positive results, the receiver operating curves were applied to identify the optimal diagnostic value. We used the Pearson *χ*^2^ test to assess any difference related to the occurrence of myosteatosis according to the pulmonary pattern.

Considering patients with muscle Hu values <30 as affected by myosteatosis [2], we used the odds-risk ratio to assess if it is associated with a higher risk of mortality.

Since the European Working Group on Sarcopenia defines muscle loss as pre-sarcopenia, and CT-based threshold values were proposed in the literature (SMI ≤ 45.471 cm^2^/m^2^ for men and ≤35.170 cm^2^/m^2^ for women), these were used to assess pre-sarcopenia in our population [13].

All analyses were performed with SPSS (v28, IBM Armonk, NY, USA) applying *P* < 0.05 as significance level.

## Results

The characteristics of the examined population are summarized in Table 1.

**Table 1.** Main characteristics of the examined population

Variable	Study population (*n* = 87)
Age (mean ± SD, years old)	60 ± 15
Gender (f:m, *n*)	77:10
Status at the last available follow-up (alive:deceased)	77:10
Height (mean ± SD, cm)	162 ± 10^a^
Weight (mean ± SD, kg)	62 ± 14^a^
Muscle area (mean ± SD, mm^2^)	67 ± 18
Muscle density (mean ± SD, Hu)	24 ± 11
Subcutaneous fat area (mean ± SD, mm^2^)	101 ± 56
Subcutaneous fat density (mean ± SD, Hu)	–89 ± 17
BMI (range, kg/m^2^)	13.9–41.1^a^
SMI (range, cm^2^/m^2^)	13.9–47.7^a^
Autoantibody positivity (*n*, %)^b^	ANA 87, 100 Anti-Scl70 37, 42.5 ACA 30, 34.5 Anti-RNA polymerase III 4,4.6 ANA without specificity 7, 8 Anti-SSA 2, 2.3 Anti-fibrillarin 1, 1.15
Limited/diffuse cutaneous disease (*n*)	53/34
mRSS (mean, range)	6 (1–39)
EUSTAR-AI (mean ± SD)	8.2 ± 8
Gastrointestinal involvement (*n*, %)	84, 96.5
Primary myocardial involvement (*n*, %)	13, 14.9
Scleroderma renal crisis (*n*, %)	1, 1.1
Interstitial lung disease (*n*, %)	65, 75
FVC predicted – (mean ± SD, %)	94 ± 24
TLC predicted – (mean ± SD, %)	86 ± 21
DLCO – (mean ± SD, %)	64 ± 20
HRCT pattern (*n*, %)	NSIP 49, 56.3 UIP 9, 10.3 OP 1, 1.1 Other 6, 6.9

a Missing data for two patients.

b Missing data for six patients

ACA: anti-centromere antibodies; ANA: antinuclear antibodies; Anti-Scl70, anti-topoisomerase I; DLCO: diffusion lung carbon monoxide; EUSTAR-AI: European Scleroderma Trials and Research Group activity index; FVC: forced vital capacity; mRSS: modified Rodnan skin score; SMI: skeletal muscle index; TLC: total lung capacity; NSIP: non-specific interstial pneumonia; UIP: usual interstitial pneumonia; OP: organizing pneumonia.

Eighty-seven patients (77 females, age range 60 ± 15 years old) were included, with a median disease duration at the time of HRCT scan of 59 (0–187) months. Thirty-four patients (39%) were treated with a low dose of steroids (prednisone ≤5 mg/day) for at least 2 years.

Sixty-five patients (75%) had signs of ILD at HRCT and the NSIP pattern was the most frequent (56.3%) (Supplementary Fig. S1, available at *Rheumatology* online). Most of the patients had lcSSc (61%) and positive anti-Scl-70 antibodies (42.5%). Thirteen patients (15%) had cardiac involvement and only one (1%) had renal crisis. Eleven patients (12.6%) were affected by pulmonary hypertension, and nine of them also had ILD. Gastrointestinal involvement was detectable in nearly all patients (*n* = 84, 97%).

The linear regression analysis demonstrated that DLCO (*P*  0.047), BMI (*P* = 0.013), density of the subcutaneous fat (*P* = 0.005) and SMI (*P* < 0.001) acted as predictors of mortality. In particular, in deceased patients there was a tendency for lower DLCO (43.6 ± 21% vs 66.3 ± 19% predicted), higher BMI (24.5 ± 5 vs 23.3 ± 4 kg/m^2^), higher density of the subcutaneous fat (–82 ± 22 vs –89 ± 16 Hu) and lower SMI values (23 ± 6 vs 25 ± 6 cm^2^/m^2^).

Patients with lcSSc were significantly older than those with dcSSc (65 ± 14 vs 53 ± 15 years old, *P* < 0.001), had lower muscle area (62 ± 13 cm^2^ vs 77 ± 24 cm^2^, *P* = 0.003), SMI (24 ± 5 vs 28 ± 7, *P* = 0.003) and more hypodense fat tissue (−92 ± 16 Hu vs −83 ± 18 Hu, *P* = 0.033) (Fig. 1c–f and Supplementary Fig. S2, available at *Rheumatology* online). As expected, lcSSc patients had lower mRSS (5 ± 3 vs 16 ± 9, *P* < 0.001) and EUSTAR-AI (1.4 ± 1.2 vs 2.4 ± 1.4, *P* < 0.001) scores.

Overall, 63 patients (72%) were affected by myosteatosis (Hu <30 Hu). Patients with myosteatosis were older (65 ± 13 vs 48 ± 15 years old, *P* < 0.001), and the age 53.5 showed 82.5% sensitivity and 70.2% specificity in detecting the presence of myosteatosis; no differences regarding disease duration occurred (*P* = 0.172). According to the thresholds proposed by Ohashi, 96% of the examined women and 89% of the men were in pre-sarcopenia [13].

The odds ratio analysis demonstrated that there was a 3.345 times higher mortality risk for patients affected by muscle fat infiltration at CT (95% CI 0.396–28.295). Patients with myosteatosis had higher mRSS scores (9 ± 8 vs 5 ± 5, *P* = 0.04), with 14 patients above the threshold value (i.e. 14), and there was a tendency for higher EUSTAR-AI scores (1.3 ± 1.3 vs 1.9 ± 1.4, *P* = 0.088), with 15 patients showing values above the 2.5 threshold.

No significant differences in body composition related to the HRCT pattern occurred (*P* = 0.081).

## Discussion

Our results suggest that function parameters and body composition variables extracted from CT images may act as predictors of mortality in patients with SSc. In particular, low SMI values and higher density of subcutaneous fat tissue showed the importance of muscle loss and inflammation on the overall outcome. In fact, even if in our study, given the retrospective study design, no histological correlations were performed, we are in line with previous evidence about other diseases and other organs (e.g. cardiac imaging) that higher attenuation values of the adipose tissue are related to higher levels of inflammation and worse clinical conditions (e.g. major cardiac events) [14]. Moreover, the results related to the DLCO confirm the systemic effect of pulmonary involvement in patients with this rare disease. Given our results, we can assume a synergistic role of the lung function impairment on body composition and vice versa. Several studies investigated such a relationship in other diseases. For example, research on 16 obese patients showed that a decrease in fat mass was associated with an improvement in lung function [15]. Another project based on MR, obtained similar results in obese young adults [16]. A study on 115 patients with fibrotic ILD showed that the pulmonary disease has a significant impact on body composition [17]. Another aspect that should be underlined is that severe gastrointestinal involvement is a predictor of mortality in SSc [18], and, given our results, the change in body composition may at least partially support this association.

Considering the lung involvement, it should also be highlighted that even if it is well known that the NSIP pattern, as also in our sample, is the most frequent in SSc, we did not find any difference in body composition metrics related to the pulmonary pattern.

In the literature, it has already been shown that the skin changes due to lcSSc may directly affect the underlying muscle and bones [19], and according to our findings, we may assume that this contributed to the reduced muscle area, SMI and more hypodense subcutaneous fat tissue in this subset of patients. Moreover, malnutrition resulting from gastrointestinal involvement, chronic inflammation, steroid therapy and decreased physical activity may be important determinants of body composition abnormalities in SSc.

Nevertheless, it should not be overlooked that these findings could also be associated with the older age of this subgroup of patients via the so-called ‘inflammaging phenomenon’ [20]. Indeed, this age-related upregulation of the immune system is connected to frailty and physical deterioration [20]. Numerous studies have already demonstrated the importance of frailty in patients with rheumatic and inflammatory diseases, including SSc, but not using imaging, at least in the latter [4, 5]. Even if the age certainly influenced our results, it should not be overlooked that patients with lcSSc had lower muscle area and SMI, suggesting that the impact of the disease on these patients should not be underestimated. In fact, although lcSSc is often considered a neglected subset, recent studies showed significant organ involvement and frequent disease manifestations with a relevant impact on quality of life in this subgroup of patients [6]. Thus, in line with recent reports, we support the need to include these patients in randomized clinical trials, possibly developing new outcome measures even based on the musculoskeletal system.

Despite the promising results, some limits affect our study. For instance, the applied threshold values for pre-sarcopenia were proposed for chronic liver diseases [13]. Projects investigating if different threshold values are needed for rheumatic and autoimmune diseases are encouraged.

Moreover, the large interval between the clinical diagnosis and the HRCT and the steroid treatment in around a third of patients cannot be overlooked. Nevertheless, these factors, did not show any significant impact, and the steroids were at a low dose. Future prospective, longitudinal studies based on the scan at diagnosis should be promoted.

Then, we relied only on CT imaging without using more advanced methods, for instance, MR-based. In fact, we applied the so-called opportunistic use of imaging which is well-known for this type of assessment and avoids additional examinations. Certainly, we call for further research providing additional insights about changes in muscle and subcutaneous tissue for example using DIXON sequences and T1 and T2 mapping.

In conclusion, patients with SSc are affected by myosteatosis and presarcopenia, and body composition seems to influence the outcome. Further prospective research including information about functional status and muscle strength is highly recommended to provide deeper insights into this preliminary evidence.

## Supplementary Material

keaf283_Supplementary_Data

## Data Availability

Data are available upon request to the corresponding author.
