# Supplementary material for: Does body composition matter in patients with systemic sclerosis?
Source: Rheumatology (Oxford). 2025 May 23;64(10):5493–7. doi: 10.1093/rheumatology/keaf283 (PMC12494221; doi:10.1093/rheumatology/keaf283)
Supplement: keaf283_Supplementary_Data [file keaf283_supplementary_data.zip › keaf283_Supplementary_Data/Supplementary material.docx]

Supplementary material

Supplementary Figure S1. Axial high-resolution chest computed tomography showing the features of non-specific interstitial pneumonia (NSIP), organizing pneumonia (OP), and usual interstitial pneumonia (UIP) patterns. In particular, in the NSIP pattern, which turned out to be the most frequent in our population, reticulations, fibrotic ground glass (yellow arrows) and traction bronchiectasis (red asterisks) in the lower lobes can be seen. In the OP pattern of a 55-year-old female patient, a peripheral irregular consolidation with reticulations is evident in the left lower lobe (white arrowhead and white arrow respectively). In the UIP pattern of a 47-year-old patient, bilateral honeycombing is easily detected (orange asterisks).

Supplementary Figure S2. Box-plots showing the differences in age, muscle area, and density of subcutaneous fat between patients with limited (lcSSc) and diffuse cutaneous disease (dcSSc).
