# Supplementary figures and images for: Does body composition matter in patients with systemic sclerosis?
Source: Rheumatology (Oxford). 2025 May 23;64(10):5493–7. doi: 10.1093/rheumatology/keaf283 (PMC12494221; doi:10.1093/rheumatology/keaf283)

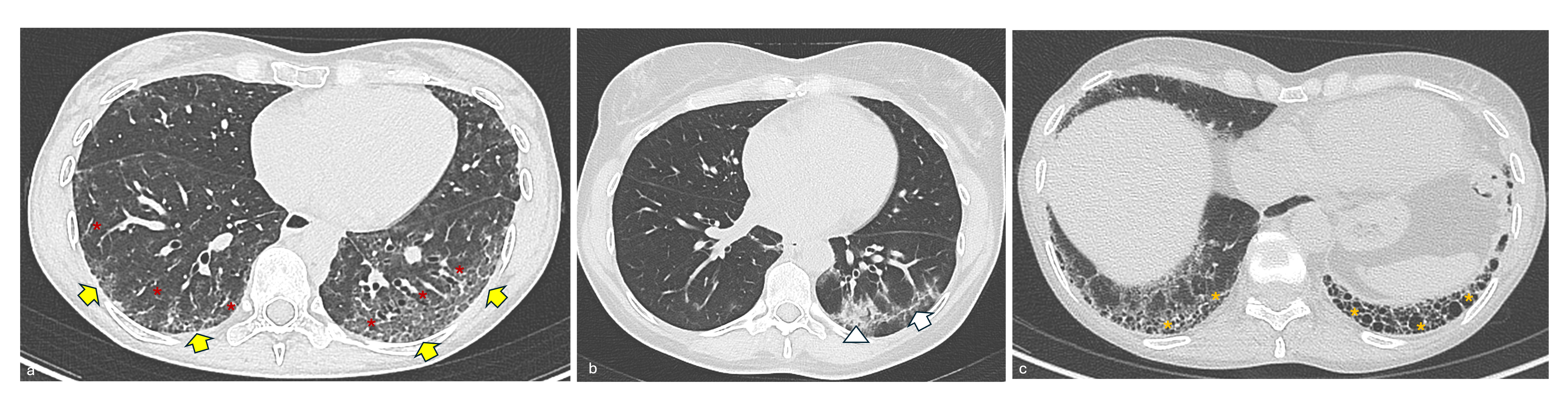

Supplement: keaf283_Supplementary_Data [file keaf283_supplementary_data.zip › keaf283_Supplementary_Data/rhe-25-0607-File004.jpg]

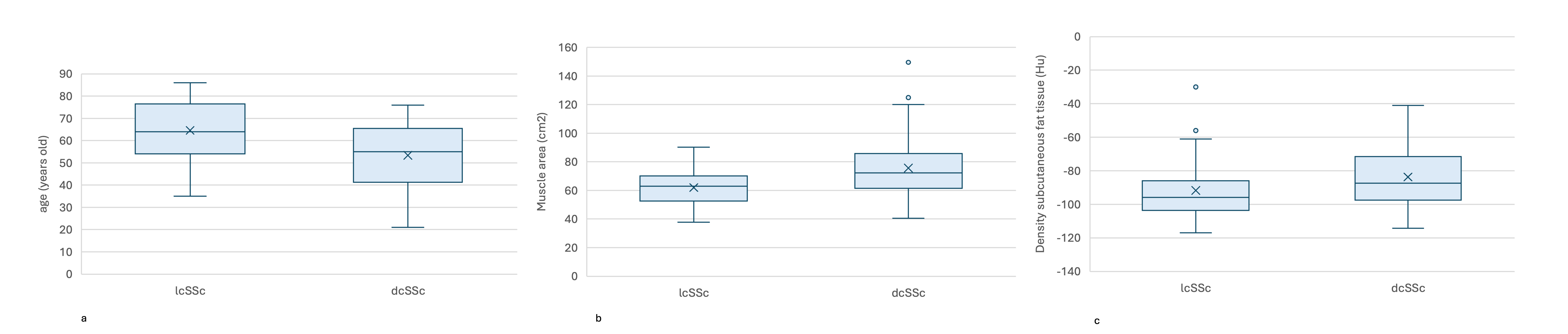

Supplement: keaf283_Supplementary_Data [file keaf283_supplementary_data.zip › keaf283_Supplementary_Data/rhe-25-0607-File005.jpg]
